# COVID-19 Vaccine Uptake and Its Impacts in a Cohort of Gay and Bisexual Men in Australia

**DOI:** 10.1007/s10461-022-03611-x

**Published:** 2022-02-08

**Authors:** G. Prestage, D. Storer, F. Jin, B. Haire, L. Maher, S. Philpot, B. Bavinton, P. Saxton, D. Murphy, M. Holt, A. Bourne, M. A. Hammoud

**Affiliations:** 1 Kirby Institute, UNSW Sydney, Wallace Wurth Building, Sydney, NSW 2052 Australia; 2 School of Population Health, University of Auckland, Auckland, New Zealand; 3 Centre for Social Research in Health, UNSW Sydney, Sydney, Australia; 4 Australian Research Centre in Sex Health & Society, La Trobe University, Melbourne, Australia

**Keywords:** Gay and bisexual men, COVID-19, Vaccines, Sexual behaviour

## Abstract

Successful use of biomedical forms of HIV risk-reduction may have predisposed many gay and bisexual men (GBM) to vaccination against COVID-19, which may, in turn, affect their sexual behavior. A total of 622 Australian GBM provided weekly data on COVID-19 vaccination history and sexual behaviour between 17 January 2021 and 22 June 2021. We identify factors associated with COVID-19 vaccination, and compare sexual behavior before and since vaccination. Mean age was 47.3 years (SD 14.0). At least one-dose vaccination coverage had reached 57.2%, and 61.3% reported that the majority of their friends intended to be vaccinated. Vaccinated men reported a mean of 1.11 (SD 2.10) weekly non-relationship sex partners before vaccination and 1.62 (SD 3.42) partners following vaccination. GBM demonstrated high confidence in COVID-19 vaccines. Their sexual activity increased following vaccination suggesting that greater sexual freedom may be a specific motivation for vaccine uptake among some men.

## Introduction

Rollout of COVID-19 vaccines is essential for controlling the pandemic, but coverage has been inconsistent in many countries [1]. People’s confidence in vaccine safety and efficacy are key to successful vaccine rollout [2]. Vulnerable populations may face additional barriers to vaccine access, with potentially inconsistent behavioral impacts [3].

In Australia, the Pfizer-BioNTech (Comirnaty TM) and the Oxford-AstraZeneca TM two-dose vaccines had received regulatory approval by mid-February 2021 [4], but their initial rollout was slow due to limited supply as well as mixed safety messaging [5, 6]. A staged approach prioritizing frontline health and essential workers, and aged and other vulnerable Australians, was implemented [6, 7].

Some people have expressed reservations about vaccination, including in Australia [2, 8]. Willingness to vaccinate appears to fluctuate over time, depending on perceived urgency and community concerns about vaccine safety [9]. Nonetheless, less than 10% of Australians have indicated they would refuse COVID-19 vaccination [2, 10].

COVID-19 vaccine rollout to priority groups, which included people living with HIV (PLHIV), officially commenced in Australia on 22 February 2021, with limited supplies of the Pfizer vaccine, and primary care rollout commenced on 22 March 2021 [6]. AstraZeneca vaccine rollout commenced on 8 March 2021. Implementation in Australia was considerably later than was the case in countries such as the United Kingdom and the United States that experienced uncontrolled epidemics [5]. Vaccines were restricted to vulnerable people, based on age, health status, and occupational status until July 2021 [4, 6]. Due to reports of thrombosis and thrombocytopenia syndrome (TTS) related to the AstraZeneca vaccine, from April 2021, Pfizer was recommended as the preferred vaccine for people under 50 years, and in June was revised to those under 60 years [4].

**Fig. 1:**
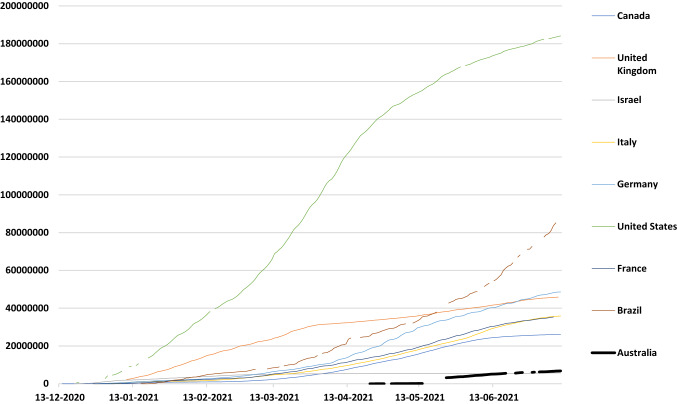
International comparisons of vaccine rollout. *Source:* https://ourworldindata.org/covid-vaccinations

By 30 June 2021, 7.65 million vaccine doses had been administered in the Australian adult population of over 22 million, representing just 30.4 doses per 100 persons, with only 144,885 individuals fully vaccinated with two doses [5, 6]. Given very different circumstances, the United Kingdom had 64.7 doses/100 persons and the United States had 69.5 doses/100 persons at equivalent stages of their vaccine rollout [6] (Fig. 1).

The pace and provision of vaccines was also slow and inconsistent at the outset, exacerbated by different requirements, changing health advice for each of the vaccine types available in Australia, and differing advice from individual state and territory health bodies [11]. Australia had a good supply of the AstraZeneca vaccine, but changing age-related recommendations [4, 6] and negative publicity about TTS impacted on willingness to take this particular vaccine [10]. With increased supply of Pfizer vaccine and increased urgency due to a growing outbreak in Sydney, vaccination rates began to improve from June 2021 [5, 6] (Table 1).

**Table 1:** Timeline: study milestones and COVID-19 pandemic responses in Australia

Date	Study milestones	COVID-19 in Australia
2014	Cohort study established	
2014–2019	Participants complete surveys at 6-monthly intervals	
January 2020		First case appears
March 2020		Initial local travel and physicial distancing restrictions imposed in each state and territory Border restrictions established, limiting international entry
April 2020	Six-monthly survey includes initial COVID-19 impact	
May 2020	Participants commence brief weekly surveys	
June 2020	New participants commence being enrolled	
February 2021		Pfizer-BioNTech (Cominarty TM) & Oxford-AstraZeneca TM receive regulatory approval COVID-19 vaccine rollout to priority groups commences
March 2021		AstraZeneca vaccine rollout commences Vaccination in primary care settings commences
April 2021		Age restrictions on AstraZeneca vaccine in response to reports of thrombosis and thrombocytopenia syndrome (TTS)
May 2021	Weekly brief surveys finish, replaced by quarterly surveys	
June 2021	First quarterly survey	7.65 million Australian adults vaccinated
July 2021		Rules restricting vaccination to only vulnerable groups eased

Resource constraints have differential impacts on access to prevention and treatment across socio-economic groups. Groups at risk of other infectious diseases, including HIV, may be particularly vulnerable to the impacts of the COVID-19 pandemic and on people’s ability to access vaccines [3, 6, 12].

Gay and bisexual men (GBM) remain at high risk for HIV through sexual contact, and gay communities have promoted behavior change to reduce that risk [13]. Initial responses to COVID-19 physical distancing restrictions in Australia saw substantial declines in sexual contacts among GBM [14]. The advent of COVID-19 vaccination may be accompanied by a return to higher levels of sexual contacts, potentially also increasing sexual risk.

The marginalized status of GBM potentially places them at elevated risk to COVID-19 outbreaks [3, 12]. They also experience more frequent sexual partnering than non-GBM and disassortative social and sexual mixing [15, 16], thereby risking rapid onward transmission of COVID-19. Also, experiences of stigma may reduce the willingness of GBM to cooperate with contact tracing efforts [12]. Alternatively, biomedical prevention interventions, including HIV pre-exposure prophylaxis (PrEP), have resulted in substantial reductions in HIV infections among GBM [17]. So, GBM may be similarly open to biomedical interventions to prevent COVID-19, including vaccines. A recent outbreak in Provincetown, Massachusetts, highlighted how tight social networks among GBM can exacerbate COVID-19 transmission, but also how the willingness of men within those networks to work with public health improved the effectiveness of contact tracing [18].

In this study, we describe uptake and types of COVID-19 vaccination among Australian GBM, examine the impact of vaccination on their sexual behavior, and investigate factors associated with vaccination.

## Methods

### Study Design and Procedure

*Following Lives Undergoing Change *(*Flux*) is an Australian national, online, open, prospective, observational cohort study of GBM, which commenced in 2014. A detailed description of the study and its protocol have been published elsewhere [14]. Eligibility criteria included: men aged at least 16 years old, who identified as gay or bisexual or had sex with a man in the previous 12 months, and lived in Australia. Enrollment was promoted online by advertising on popular social media platforms and through gay community and HIV community organisations around Australia. Prior to 2020, participants completed surveys at 6-month intervals. Commencing in May 2020, participants were invited to complete brief weekly surveys to monitor the impacts of COVID-19. New participants were recruited starting in week 8 of the study (28 June 2020). Participants provided online informed consent, and enrollment was verified upon activating a link via email. Ethical approval was granted from UNSW Sydney.

### Measures

Demographic items were collected at baseline. The brief weekly questionnaire captured data about sexual behavior, social connectedness, and access to healthcare in weeks 1–52 (10 May 2020 to 2 May 2021), and again at week 59 (23–30 June 2021). This included questions about testing for COVID-19 during the previous week. Sexual behavior questions included sex with non-relationship partners, either non-romantic regular partners or casual partners [19]. Questions about COVID-19 vaccination were introduced in week 37 (23 January 2021) prior to the vaccine roll out in Australia. Men were also asked additional questions once every 4 weeks, including: beliefs about COVID-19 vaccines; intention to vaccinate; and proportion of their friends they believed intended to be vaccinated.

### Participants

Between weeks 37 and 52, 547 men responded to the weekly surveys including 141 who were newly enrolled in 2020. An additional 121 men from the ongoing Flux cohort also responded in week 59, making a total of 668 respondents between weeks 37 and 59. All but 46 of these men responded to questions about COVID-19 vaccination, making a total sample of 622 men included in these analyses.

### Analysis

#### Vaccination Status

We present trends in weekly and cumulative vaccine uptake between weeks 37 and 59, and describe differences between men who had been vaccinated (with at least one dose) by week 59 and men who had not yet been vaccinated. Descriptive statistics were used to characterize men according to their reported vaccination status by week 59. Factors associated with vaccination were identified in bivariate analysis with t-tests for continuous variables and chi-square tests for categorical variables. In multivariable analysis, factors independently associated with vaccination were identified using logistic regression; we present adjusted odds ratios (aOR), 95% confidence intervals (95% CI) and p-values.

#### Changes in Sexual Behavior

In separate analyses, accounting for several weeks without data collection between weeks 52 and 59, we excluded men who had not received at least one vaccine dose by week 59, and compared sex with non-relationship sexual partners for the week that fell at least 12 weeks prior to vaccination with sex with partners in their most recent week following vaccination. Paired t-tests were conducted to compare mean number of partners before and since COVID-19 vaccination using a Type 1 error rate of 5%. To examine changes in sexual contact from before and since vaccination, we used the McNemar method for nonparametric tests of two related samples. Data were analysed using SPSS™ version 26 software.

## Results

Compared to the 622 men who responded to questions about vaccination in the weekly surveys between weeks 37 and 59, the 46 men who did not respond to vaccination questions did not differ on any key demographic or behavioral variables other than age. These 46 men excluded from analyses were younger: mean age was 40.6 years (SD 13.1) vs 47.3 years (SD 14.0) among the 622 men included in this sample (OR 1.04; 95% CI 1.01–1.06; p = 0.003).

Most of the 622 men in this sample were born in Australia (76.8%) or New Zealand (3.3%). Over half lived in NSW (44.4%) or Victoria (24.9%), and over a third (34.9%) lived in postcodes where at least 5% of the adult male population identified as gay [20]. Most had completed either undergraduate (33.9%) or postgraduate (36.3%) university education and were in either full-time (60.9%) or part-time (14.3%) employment. Most (95.5%) had been tested for HIV, with 9.2% indicating they were HIV-positive. Most men identified as either gay (89.5%) or bisexual (6.4%), and 83.6% reported sex with men during the study period. About three quarters (72.2%) reported sex with any non-relationship partners during the study period. Men reported having a mean of 14.7 gay friends (median = 10; SD 23.03), and 35.1% indicated that most of their friends were gay men. Most recently, men reported close physical contact with a mean of 9.68 people (median = 5; SD 19.95) in the previous week. Nearly two thirds (61.3%) believed the majority of their friends intended to be vaccinated against COVID-19; 28.0% believed that fewer than half their friends, and 7.4% that none of their friends intended to be vaccinated. Nearly half (47.9%) reported that at least half their friends had been vaccinated by week 59.

Overall, 62.1% of men had been tested for COVID-19, with the majority of those tested once (26.2%) or twice (12.2%). On average, about 4% had been tested each week (Table 2). Notably, men in professional or managerial occupations had been tested more often (mean = 1.92; SD 2.39) than other men (mean = 1.38; SD 2.26; OR 1.24; 95% CI 1.08–1.43; p = 0.002).

**Table 2:** Numbers and proportions of GBM responding each study week who were tested for COVID-19 and who received COVID-19 vaccinations. (N = 622)

Date	Week number	Number of responses	Proportion tested for COVID-19 in previous 7 days	Number of initial vaccinations reported	Proportion of all vaccinations reported (N = 347)	Cumulative number of initial vaccinations reported	Cumulative proportion vaccinated
10-May-20 to 10-Jan-21	1–36	570	NA	0	0.0%	0	0.0%
17-Jan-21	37	417	4.3%	2	0.6%	2	0.3%
24-Jan-21	38	429	4.4%	0	0.0%	2	0.3%
31-Jan-21	39	438	3.0%	0	0.0%	2	0.3%
07-Feb-21	40	437	5.3%	0	0.0%	2	0.3%
14-Feb-21	41	439	3.4%	0	0.0%	2	0.3%
21-Feb-21	42	442	3.2%	0	0.0%	2	0.3%
28-Feb-21	43	431	2.3%	3	0.9%	5	0.8%
07-Mar-21	44	441	3.6%	3	0.9%	8	1.3%
14-Mar-21	45	429	4.0%	4	1.2%	12	2.0%
21-Mar-21	46	435	2.5%	7	2.0%	19	3.2%
28-Mar-21	47	423	4.3%	18	5.2%	37	6.1%
4-Apr-21	48	427	6.3%	18	5.2%	55	9.1%
11-Apr-21	49	424	4.3%	11	3.2%	66	10.9%
18-Apr-21	50	424	4.5%	14	4.0%	80	13.3%
25-Apr-21	51	423	2.6%	13	3.7%	93	15.4%
2-May-21	52	410	4.4%	13	3.7%	106	17.6%
22-Jun-21	59	536	5.6%*	34*	9.6%*	356	57.2%

*Represents a mean value across the intervening 7 weeks between weeks 52 and 59. Number of actual initial vaccinations between weeks 52 and 59 was 250, representing 70.2% of all vaccinations received

**Fig. 2:**
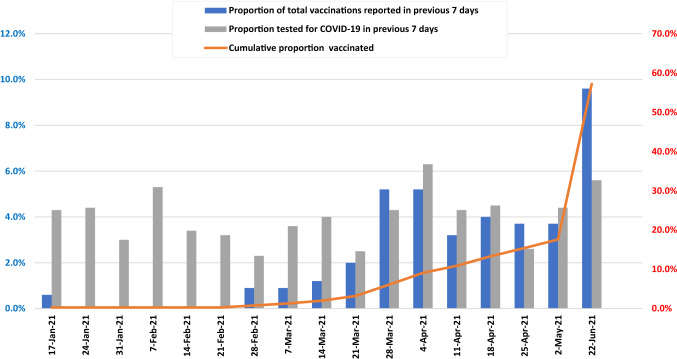
Weekly and cumulative vaccination rates (N = 622)

In week 37, prior to the start of the public vaccine rollout, two men reported having already been (fully) vaccinated, both through clinical trials (Table 2). No others reported receiving vaccination until week 43. Thereafter, numbers who reported vaccination increased, until by week 52, 106 (17.6%) men had been vaccinated with at least one dose (Fig. 2). Between week 52 and week 59, another 250 men had been vaccinated, resulting in an overall vaccine coverage (at least one dose) of 57.2%.

Men without university education, younger men, and men in non-professional or non-managerial occupations were less likely to have been vaccinated (Table 3). State of residence, country of birth, employment status, HIV serostatus, and sexual identity were not associated with vaccination.

**Table 3:** Characteristics of sample according to whether vaccinated or not vaccinated. (N = 622)

N (%)	Unvaccinated (n = 266)	Vaccinated (n = 356)	OR (95% CI)	p-value	aOR (95% CI)	p-value
Age–mean (SD)	**41.25 (12.44)**	**51.71 (13.49)**	**1.06 (1.05–1.08)**	** < 0.001**	**1.07 (1.05–1.09)**	**< 0.001**
16–30	55 (64.7)	30 (35.3)				
31–40	91 (64.5)	50 (35.5)				
41–50	52 (40.9)	75 (59.1)				
51–60	44 (32.8)	90 (67.2)				
Over 60	19 (15.1)	107 (84.9)				
No data	5	4				
Education						
Postgraduate	80 (35.4)	146 (64.6)	1.00			
Undergraduate	99 (46.9)	112 (53.1)	0.63 (0.42–0.91)	0.015		
Not university-level	84 (46.7)	96 (53.3)	0.62 (0.42–0.93)	0.022		
No data	3	2				
Employment status						
Full-time employed	169 (44.6)	210 (55.4)	1.00			
Part-time employed	42 (47.2)	47 (52.8)	0.68 (0.44–1.03)	0.068		
Not in workforce	55 (35.7)	99 (64.3)	0.69 (0.47–1.01)	0.058		
Occupation						
Profession/manager	147 (41.4)	208 (58.6)	1.00			
Other	64 (56.6)	49 (43.4)	0.54 (0.35–0.83)	0.005		
Not in workforce	55 (35.7)	99 (64.3)	1.27 (0.86–1.88)	0.228		
State						
NSW	123 (44.6)	153 (55.4)	1.00			
Victoria	61 (39.4)	94 (60.6)	1.24 (0.83–1.85)	0.294		
Queensland	31 (38.3)	50 (61.7)	1.30 (0.78–2.15)	0.315		
Other states/territories	43 (46.2)	50 (53.8)	0.88 (0.54–1.43)	0.613		
No data	8	9				
Country of birth						
Australia/New Zealand	210 (42.1)	289 (57.9)	1.00			
Elsewhere	42 (41.2)	60 (58.8)	1.04 (0.67–1.60)	0.866		
No data	14	7				
Sexual identity						
Gay	232 (41.7)	325 (58.3)	1.00			
Other	27 (50.9)	26 (49.1)	0.69 (0.39–1.21)	0.193		
No data	7	5				
HIV serostatus						
HIV-negative	227 (42.3)	310 (57.7)	1.00			
HIV-positive	22 (38.6)	35 (61.4)	1.17 (0.67–2.04)	0.593		
Unknown/untested	17 (60.7)	11 (39.3)	0.47 (0.22–1.03)	0.060		
Influenza vaccination						
Not vaccinated	112 (53.8)	96 (46.2)	1.00			
Has been vaccinated	154 (37.2)	260 (62.8)	1.97 (1.41–2.76)	< 0.001		
Sexual contact with non-relationship partners in most recent week						
No non-relationship partners	153 (45.3)	185 (54.7)	1.00			
Any non-relationship partners	104 (39.2)	161 (60.8)	1.28 (0.92–1.78)	0.138		
No data	9	10				
COVID-19 test number—mean (SD)	**1.39 (1.84)**	**1.90 (2.70)**	**1.11 (1.02–1.19)**	**0.010**	**1.15 (1.05–1.25)**	**0.002**
Never tested	108 (45.8)	128 (54.2)				
Ever tested	158 (40.9)	228 (59.1)				
Beliefs about how long COVID-19 restrictions would be needed						
Longer than a year	99 (35.2)	182 (64.8)	1.00		1.00	
About 1 year	112 (44.4)	140 (55.6)	0.68 (0.48–096)	0.030	0.83 (0.55–1.25)	0.370
Less than 1 year	55 (61.8)	34 (38.2)	0.34 (0.21–0.55)	< 0.001	0.36 (0.21–0.64)	< 0.001
Number of friends intending to be vaccinated						
None/less than half	124 (56.4)	96 (43.6)	1.00			
Majority	132 (34.6)	249(65.4)	2.44 (1.73–3.42)	< 0.001		
No data	10	11				
“I am worried about side effects”—mean (SD)	**2.36 (1.57)**	**1.66 (1.38)**	**0.73 (0.65–0.81)**	** < 0.001**	**0.84 (0.72–0.98)**	**0.026**
Strongly disagree	42 (32.3)	88 (67.7)				
Disagree	51 (32.5)	106 (67.5)				
Slightly disagree	31 (44.3)	39 (55.7)				
Slightly agree	80 (46.5)	92 (53.5)				
Agree	34 (59.6)	23 (40.4)				
Strongly agree	28 (77.8)	8 (22.2)				
“Vaccines are too new to be confident”—mean (SD)	**1.53 (1.58)**	**0.74 (0.98)**	**0.62 (0.54–0.71)**	** < 0.001**	**0.66 (0.55–0.79)**	** < 0.001**
Strongly disagree	84 (31.3)	184 (68.7)				
Disagree	86 (42.8)	115 (57.2)				
Slightly disagree	29 (50.0)	29 (50.0)				
Slightly agree	29 (58.0)	21 (42.0)				
Agree	15 (71.4)	6 (28.6)				
Strongly agree	23 (95.8)	1 (4.2)				
Number of friends & family spent physical time with in previous 7 days—mean (SD)	**9.76 (22.61)**	**9.62 (17.72)**	**1.00 (0.99–1.01)**	**0.938**		

Bold values describe statistics for variable, with specific response categories in following rows

Having more friends that they believed intended to be vaccinated was associated with vaccine uptake (Table 3). Sexual behavior and spending physical time with friends and family were not associated with vaccination.

Having received an influenza vaccination during 2020–2021 and having been tested multiple times for COVID-19 were associated with receiving the COVID-19 vaccination (Table 3). Men who expressed concerns about vaccine side effects (43.8%) or the speed of their development (14.7%) were less likely to have been vaccinated (Table 3).

In multivariable analysis, older age, having been tested more often for COVID-19, expecting COVID-19 restrictions to remain necessary for more than a year, and being less sceptical about COVID-19 vaccines were associated with having been vaccinated.

Restricting the sample to the 356 men who had been vaccinated by week 59, nearly half of those vaccinated by week 52 but only one quarter of those vaccinated after week 52 had received their second dose of the vaccine. Three quarters of those who had been vaccinated by week 52 had received the AstraZeneca vaccine and most others had received the Pfizer vaccine (Table 4). Although the majority of those who were vaccinated between weeks 52 and 59 had received the AstraZeneca vaccine, participants vaccinated during this period were more likely to have received the Pfizer vaccine than were those who had vaccinated earlier. Half (50.4%) of those who had received the AstraZeneca vaccine, 25.8% of those who received the Pfizer vaccine, and 41.8% of unvaccinated men indicated that they resented not having a choice of vaccine (χ^2^ = 48.98; p = 0.003).

**Table 4:** Type and dosage of vaccine received and number of sex partners before and since vaccination according to date of vaccination. (N = 356)

	Vaccinated by May 2nd 2021 N = 106	Vaccinated between May 3rd and June 30th 2021 N = 250	χ^2^	p-value
Vaccination type			**17.85**	**0.001**
AstraZeneca	78 (73.6)	136 (54.4)		
Pfizer	26 (24.5)	113 (45.2)		
Novavax/covax	1 (0.9)	1 (0.4)		
Unknown	1 (0.9)	0 (0.0)		
Number of doses (by week 59)			**9.95**	**0.001**
First dose only	61 (57.5)	186 (74.4)		
Second dose	45 (42.5)	64 (25.6)		

Mean number of non-relationship partners			OR (95% CI)	p-value
12 weeks before vaccination	1.04 (1.87)	1.14 (2.21)	1.02 (0.91–1.15)	0.700
Most recent week since vaccination	1.67 (4.06)	1.59 (3.03)	0.99 (0.93–1.06)	0.847

Bold values describe statistics for variable, with specific response categories in following rows

Whereas by week 59, only slightly more HIV-positive men had been vaccinated than had other men, HIV-positive men did appear to have been vaccinated earlier. At week 52, 40.0% of vaccinations among HIV-positive men had already occurred as had 28.6% of vaccinations among other men (non-significant).

Among men who had received their vaccination, men reported a mean of 1.11 (SD 2.10) non-relationship sexual partners for the week that fell at least 12 weeks prior to vaccination and a mean of 1.62 (SD 3.42) partners in their most recent week following vaccination [t(256) = 2.18; p = 0.030]. The majority of men (54.5%) did not have sex with any non-relationship partners prior to vaccination, but this had fallen to 46.7% since vaccination (χ^2^ = 5.16; p = 0.023). Also, whereas 55.0% of unvaccinated men agreed that vaccination would make them feel safer to have sex with casual partners, this was true of 64.9% of vaccinated men (χ^2^ = 5.14; p = 0.015).

Among 191 men who responded to the survey in week 59 and who remained unvaccinated, 51 (26.7%) had made an appointment to be vaccinated, 43 of whom were scheduled to receive the Pfizer vaccine. Of the 140 men who had not made an appointment to be vaccinated, 52.9% indicated that they had not done so because they were not yet eligible and 15.0% said they could not access vaccination. Nonetheless, most of those who had not yet been vaccinated (83.2%) indicated they were likely to be vaccinated when they were able to do so. Just eight men indicated they would not be vaccinated and 15 men were waiting until they could access the vaccine of their choice.

At week 59, 117 men reported sex with non-relationship partners, 86 of whom had been vaccinated. Among these 86 vaccinated men, 93.0% indicated they would be very willing to contact these sex partners if needed for COVID-19 contact tracing, as did 77.4% of the 31 men who were unvaccinated (χ^2^ = 8.20; p = 0.017).

Beliefs about the relative effectiveness and impacts of vaccines were similar between men vaccinated by week 52 and those vaccinated later (Table 5). Unvaccinated men were somewhat more sceptical.

**Table 5:** Beliefs about effectiveness, safety, and impact of COVID-19 vaccines at week 59. (N = 536)

	Vaccinated by May 2nd 2021 N = 95	Vaccinated between May 3rd and June 30th 2021 N = 250	Unvaccinated (N = 191)	χ^2^	p-value
“AstraZeneca and Pfizer are equally effective”				**14.92**	**0.001**
Disagree	38 (40.0)	77 (31.2)	92 (49.5)		
Agree	57 (60.0)	170 (68.8)	94 (50.5)		
No data	0	3	5		
“I doubt vaccine safety”				**17.80**	** < 0.001**
Disagree	80 (84.2)	210 (85.1)	131 (69.3)		
Agree	15 (15.8)	37 (14.9)	58 (30.8)		
No data	0	3	2		
“After vaccines we can forget about COVID”				**2.39**	**0.303**
Disagree	82 (86.3)	203 (82.3)	165 (87.3)		
Agree	13 (13.7)	44 (17.7)	24 (12.7)		
No data	0	3	2		
“Having vaccine will make following feel safer”					
Being out in public	86 (90.5)	224 (89.6)	149 (78.0)	**14.07**	**0.001**
Using public transport	82 (86.3)	213 (85.2)	146 (76.4)	**6.99**	**0.030**
Having casual sex	58 (61.1)	166 (66.4)	105 (55.0)	**5.97**	**0.051**

Bold values describe statistics for variable, with specific response categories in following rows

There was no association between brand of vaccine received and beliefs about the relative effectiveness or impacts of COVID-19 vaccines.

## Discussion

Whereas by the end of the study period, in late June 2021, less than 35% of adult Australians had received at least a single dose of COVID-19 vaccination [2], in this sample, it was nearly 60%. Furthermore, most men expressed confidence that COVID-19 vaccines were safe and effective, and those who remained unvaccinated mostly intended to be vaccinated when that option became available to them, demonstrating little evidence of vaccine hesitancy.

Men in this sample tended to have high socioeconomic status, and were somewhat older in age, both of which are also associated with willingness to vaccinate in the Australian population [9] They may have simply had better access to health care in general, and more opportunities for vaccination specifically. Also, similar to our own findings, unwillingness to be vaccinated appears to be less than 10% of Australians [2, 10], and associated with lower education [9].

More frequent testing for COVID-19 was associated with vaccination. Some men may have been working in high risk professions, such as healthcare, as suggested by the greater frequency of testing among men in professional or managerial occupations, and their greater likelihood to have been vaccinated. These men’s need to test may itself have encouraged them to seek vaccination. As has been found elsewhere, recent influenza vaccination was also associated with COVID-19 vaccination [21]. Greater engagement with health care in general may also indicate greater understanding of the importance of vaccination.

Most men in this sample were gay-identified and strongly embedded in gay community life, both where they live and through their friendship networks. Past experience with HIV and with biomedical forms of HIV prevention [13, 17] may have facilitated greater confidence with health systems in general and with medical interventions to prevent disease specifically. This may explain the expressed willingness of most men to assist with COVID-19 contact tracing if required, particularly those who had been vaccinated, similar to what occurred during an outbreak among GBM in the United States [18]. Further research is warranted into willingness to assist with contact tracing among men who have sex with men but who are not strongly engaged in gay community life.

The association of vaccination with having more friends who intended to be vaccinated suggests that peer norms play a role in vaccination uptake. In other settings, identification with tight social networks has been associated with COVID-19 vaccination uptake and facilitates adherence to mutual protection [22, 23]. Individuals who demonstrate specific health-seeking and protective behaviors undoubtedly encourage similar behaviors within their peer networks, thereby normalizing such behaviors. For GBM, gay social engagement has previously been associated with HIV testing [24], and with uptake of PrEP [25], becoming normalized behaviors, particularly in relation to reducing risk of HIV transmission during sex. For GBM, gay community networks offer supportive frameworks in responding to the challenges of COVID-19 [26], and may be rapidly normalizing COVID-19 vaccination as well, and perhaps with at least some similar motivations.

Uptake of PrEP among GBM has been accompanied by increased sexual activity and reduced anxiety about HIV transmission [27, 28]. COVID-19 vaccination also precipitated a subsequent 45% increase in non-relationship partners in this sample. During the initial response to the COVID-19 pandemic, Australian GBM had dramatically reduced their sexual contacts and their social interactions [14]. Many vaccinated men indicated that vaccination would make them feel safer to have sex with casual partners. So, vaccination may encourage some men to recommence a more active sex life; increased sexual concurrency might also represent increased risks for viral transmission [29]. However, this will undoubtedly depend on future outbreaks and accompanying restrictions, and the extent to which GBM utilize risk-reduction techniques and biomedical prevention. Future research should investigate the extent to which COVID-19 vaccination has permitted GBM to maintain active sex lives regardless of ongoing changes in the pandemic.

Although PLHIV are a designated priority group for COVID-19 vaccination [6], HIV-positive men were not significantly more likely to have vaccinated than HIV-negative men. Nonetheless, there was a slight tendency for them to have vaccinated somewhat earlier than other men in the sample.

The finding that younger men were less likely to have been vaccinated is unsurprising. Initially, older people were prioritized for vaccination in Australia, and the AstraZeneca vaccine was restricted to older persons [4], with limited supply of Pfizer. Nonetheless, further research into the response to COVID-19 among younger GBM would be warranted.

We found no differences in levels of scepticism between men who had received the AstraZeneca vaccine and those who had received the Pfizer vaccine. However, the process for the rollout of vaccines across Australia has meant that most people have little choice as to which vaccine they receive. So, higher levels of scepticism regarding specific vaccine types may have applied regardless of which vaccine they were eventually offered. That men who had received the AstraZeneca vaccine were more likely to resent their lack of vaccine choice suggests this might be the case.

Whereas most men who had been vaccinated had received the AstraZeneca vaccine, it was notable that those who made an appointment to be vaccinated in the near future had arranged to receive the Pfizer vaccine. Despite being generally confident of the safety and effectiveness of COVID-19 vaccines in general, and expressing faith in both vaccine types, it is likely that ongoing coverage in the Australian media of the increased risk of TTS associated with the AstraZeneca vaccine may nonetheless be contributing to a preference to receive the Pfizer vaccine.

### Limitations

Findings from this online convenience sample may not be representative of all GBM men in Australia; the sample was somewhat older and more highly educated than other typical samples of Australian GBM [20]. Measures related to experiences of COVID-19 were not validated, but due to the rapid onset of COVID-19 and the desire to implement rapid monitoring, this was unavoidable and likely affects most early COVID-19 research studies. Data collected from the period before COVID-19 were only available for those men who had been previously enrolled into the cohort, during 2014–2018. The rollout of COVID-19 vaccines in Australia continues to change rapidly, so findings reported here may be specific to the time period covered (May 2020 to June 2021). Subsequent outbreaks, such as the large Sydney outbreak that occurred following the data collection period, are not reflected in these data. Some men who first reported their vaccination in week 59 may have also been vaccinated in that same week and so the sexual behavior reported would have been during the same week as their vaccination. Analyses were constrained by the small numbers of HIV-positive men in the sample.

## Conclusions

Most Australian GBM in this sample had confidence in the safety and efficacy of COVID-19 vaccines and over half had received at least one dose. Most of those not yet vaccinated intended to do so, as and when they can. Younger men were less likely to have been vaccinated, but this most likely reflected vaccine eligibility criteria in Australia in a context of vaccine scarcity. Scepticism about COVID-19 vaccines was a barrier to vaccine uptake. Social connection, including with peers who intended to be vaccinated, may have encouraged some men to seek vaccination, perhaps particularly among those motivated to become more sexually active.

## Data Availability

Data are available on request to the authors.

## Code Availability

SPSS version 26 was used for data analysis.

## References

[1] Burki TK (2021). Challenges in the rollout of COVID-19 vaccines worldwide. Lancet Respir Med.

[2] Dodd RH, Cvejic E, Bonner C, Pickles K, McCaffery KJ, Ayre J (2021). Willingness to vaccinate against COVID-19 in Australia. Lancet Infect Dis.

[3] Iversen J, Sabin K, Chang J, Morgan Thomas R, Prestage G, Strathdee SA (2020). COVID-19, HIV and key populations: cross-cutting issues and the need for population-specific responses. J Int AIDS Soc.

[4] Australian Government Department of Health Therapeutic Goods Administration. COVID-19 vaccines. https://www.tga.gov.au/covid-19-vaccines. Accessed 5 Sept 2021.

[5] Our World in Data. Statistics and research: coronavirus pandemic (COVID-19). https://ourworldindata.org/coronavirus. Accessed 6 Sept 2021.

[6] Australian Government Department of Health. COVID-19 vaccines. https://www.health.gov.au/initiatives-and-programs/covid-19-vaccines. Accessed 5 Sept 2021.

[7] MacIntyre CR, Costantino V, Trent M (2021). Modelling of COVID-19 vaccination strategies and herd immunity, in scenarios of limited and full vaccine supply in NSW, Australia. Vaccine.

[8] Rhodes A, Hoq M, Measey MA, Danchin M (2021). Intention to vaccinate against COVID-19 in Australia. Lancet Infect Dis.

[9] Essential Research. The essential report (2021). https://essentialvision.com.au/wp-content/uploads/2021/08/Essential-Report-160821_V2.pdf. Accessed 29 Aug 2021.

[10] Dong E, Du H, Gardner L (2020). An interactive web-based dashboard to track COVID-19 in real time. Lancet Infect Dis.

[11] Reynolds G, Trevillyan JM (2021). Australia’s COVID-19 vaccination program. Respir Med Today.

[12] Gibb JK, DuBois LZ, Williams S, McKerracher L, Juster RP, Fields J (2020). Sexual and gender minority health vulnerabilities during the COVID-19 health crisis. Am J Hum Biol.

[13] Dowsett GW (2009). Dangerous desires and post-queer HIV prevention: rethinking community, incitement and intervention. Soc Theory Health.

[14] Hammoud MA, Maher L, Holt M, Degenhardt L, Jin F, Murphy D (2020). Physical distancing due to COVID-19 disrupts sexual behaviours among gay and bisexual men in Australia. J Acquir Immune Defic Syndr.

[15] Glick SN, Morris M, Foxman B, Aral SO, Manhart LE, Holmes KK (2012). A comparison of sexual behavior patterns among men who have sex with men and heterosexual men and women. J Acquir Immune Defic Syndr.

[16] Smith AM, Richters J, Rissel CE, Grulich AE, de Visser RO, Badcock PB, Temple-Smith M (2014). The sexual practices of theAustralian population. Sexual Health: A multidisciplinary approach.

[17] Grulich AE, Jin F, Bavinton BR, Yeung B, Hammoud MA, Amin J (2021). Long-term protection from HIV infection with oral HIV pre-exposure prophylaxis in gay and bisexual men: findings from the expanded and extended EPIC-NSW prospective implementation study. Lancet HIV.

[18] Marks A. What Provincetown’s gay community can teach us about containing covid. The Rolling Stone (2021). https://www.rollingstone.com/culture/culture-features/covid-delta-variant-provincetowns-gay-community-1214665/. Accessed 4 Sept 2021.

[19] Bavinton BR, Duncan D, Grierson J, Zablotska IB, Down IA, Grulich AE (2016). The meaning of ‘regular partner’in HIV research among gay and bisexual men: implications of an Australian cross-sectional survey. AIDS Behav.

[20] Callander D, Mooney-Somers J, Keen P, Guy R, Duck T, Bavinton B (2020). Australian ‘gayborhoods’ and ‘lesborhoods’: a new method for estimating the number and prevalence of adult gay men and lesbian women living in each Australian postcode. Int J Geogr Inf Sci.

[21] Wang Q, Yang L, Jin H, Lin L (2021). Vaccination against COVID-19: a systematic review and meta-analysis of acceptability and its predictors. Prev Med.

[22] Wakefield JRH, Khauser A (2021). Doing it for us: community identification predicts willingness to receive a COVID-19 vaccination via perceived sense of duty to the community. J Commun Appl Soc Psychol.

[23] Stevenson C, Wakefield JRH, Felsner I, Drury J, Costa S (2021). Collectively coping with coronavirus: local community identification predicts giving support and lockdown adherence during the COVID-19 pandemic. Br J Soc Psychol.

[24] Flowers P, Knussen C, Li J, McDaid L (2013). Has testing been normalized? An analysis of changes in barriers to HIV testing among men who have sex with men between 2000 and 2010 in Scotland, UK. HIV Med.

[25] Hammoud MA, Vaccher S, Jin F, Bourne A, Maher L, Holt M (2019). HIV pre-exposure prophylaxis (PrEP) uptake among gay and bisexual men in Australia and factors associated with the non-use of PrEP among eligible men. J Acquir. Immune Defic Ssyndr.

[26] Philpot SP, Holt M, Murphy D, Haire B, Prestage G, Maher L (2021). Qualitative findings on the impact of COVID-19 restrictions on Australian gay and bisexual men: community belonging and mental well-being. Qual Health Res.

[27] Prestage G, Maher L, Grulich A, Bourne A, Hammoud M, Vaccher S (2019). Brief report: changes in behavior after PrEP initiation among Australian gay and bisexual men. J Acquir Immune Defic Syndrome.

[28] Keen P, Hammoud MA, Bourne A, Bavinton BR, Holt M, Vaccher S (2020). Use of HIV pre-exposure prophylaxis (PrEP) associated with lower HIV anxiety among gay and bisexual men in Australia who are at high risk of HIV infection: results from the Flux Study. J Acquir Immune Defic Syndr.

[29] Morris M, Goodreau S, Moody J (2008). Sexual networks, concurrency, and STD/HIV. Sex Transm Dis.

